# Risks of mental health of foreign medical residents who study in Ukraine

**DOI:** 10.1192/j.eurpsy.2024.563

**Published:** 2024-08-27

**Authors:** M. Markova, A. Kaafarani, T. Aliieva

**Affiliations:** ^1^Kharkiv National Medical University, Kharkiv, Ukraine

## Abstract

**Introduction:**

The high level of psycho-emotional stress today significantly increases the moral burden on the mental health of medical workers. One of the most vulnerable groups of medical specialists in Ukraine are foreign resident doctors. Since in Ukraine this contingent of doctors is faced with extraordinary problems of high psycho-emotional stress, such as COVID-19 around the world and the russian war against Ukraine. Raising the issue of resident doctors, the main issue becomes not only their professional identity, but also the formation of the necessary stress resistance in different conditions of professional activity.

**Objectives:**

To study the level of tolerance to stress and uncertainty among foreign resident doctors in unusual conditions of performing professional duties.

**Methods:**

The examination included the use of clinical-psychological, psychodiagnostic and psychometric research methods.

**Results:**

As of the beginning of 2020, 395 foreigners were studying. During the first phase of the pandemic, 118 foreigners left Ukraine. By the beginning of 2022 (before the full-scale war), 302 medical residents were trained. As of the beginning of 2023, 167 doctors are studying, of which only 61 people are on the territory of Ukraine.

The primary analysis of the clinical-psychological study showed that the most common complaints among foreign resident doctors are: increased levels of feelings of tension (in 75,4%), decreased motivation for activity (73,2%), anxious (72,7%) and depressive symptoms (69,3%), frequent headaches (68.6%), constant feelings of irritation (65,4%), manifestations of aggression in relation to colleagues (63,9%) and patients(61,4%), a feeling of fear for the future (60,1%), conflicts in the family (59,5%).

The study of factors that influence the increase in the level of stress among foreign resident doctors were sorted according to the principle of ordinary and extraordinary. Ordinary stress factors include: the nature of the specialty, the conditions of professional activity, a foreign country, relationships in the team. Extraordinary factors include new working conditions (professional challenges of COVID-19, war on the territory of Ukraine), increased risks of responsibility for the patient’s life (search for a treatment solution against the background of COVID-19), nature of assistance (providing assistance due to combat injuries).

**Conclusions:**

At the end of the study, a comprehensive program will be created for the early detection of signs of adaptation disorder, which will be aimed at reducing emotional distress, tension in the learning process, support in the first years of training for medical residents.

**Disclosure of Interest:**

None Declared

